# Prediction of clinical toxicity in localized cervical carcinoma by radio-induced apoptosis study in peripheral blood lymphocytes (PBLs)

**DOI:** 10.1186/1748-717X-4-58

**Published:** 2009-11-26

**Authors:** Elisa Bordón, Luis Alberto Henríquez Hernández, Pedro C Lara, Beatriz Pinar, Fausto Fontes, Carlos Rodríguez Gallego, Marta Lloret

**Affiliations:** 1 Canary Institute for Cancer Research (ICIC), Las Palmas, Spain; 2 Clinic Sciences Department of Las Palmas de Gran Canaria University (ULPGC), Las Palmas, Spain; 3 Radiation Oncology Department, Hospital Universitario de Gran Canaria Dr. Negrín, Las Palmas, Spain; 4 Inmunology Department, Hospital Universitario de Gran Canaria Dr. Negrín, Las Palmas, Spain

## Abstract

**Background:**

Cervical cancer is treated mainly by surgery and radiotherapy. Toxicity due to radiation is a limiting factor for treatment success. Determination of lymphocyte radiosensitivity by radio-induced apoptosis arises as a possible method for predictive test development. The aim of this study was to analyze radio-induced apoptosis of peripheral blood lymphocytes.

**Methods:**

Ninety four consecutive patients suffering from cervical carcinoma, diagnosed and treated in our institution, and four healthy controls were included in the study. Toxicity was evaluated using the Lent-Soma scale. Peripheral blood lymphocytes were isolated and irradiated at 0, 1, 2 and 8 Gy during 24, 48 and 72 hours. Apoptosis was measured by flow cytometry using annexin V/propidium iodide to determine early and late apoptosis. Lymphocytes were marked with CD45 APC-conjugated monoclonal antibody.

**Results:**

Radiation-induced apoptosis (RIA) increased with radiation dose and time of incubation. Data strongly fitted to a semi logarithmic model as follows: RIA = βln(Gy) + α. This mathematical model was defined by two constants: α, is the origin of the curve in the Y axis and determines the percentage of spontaneous cell death and β, is the slope of the curve and determines the percentage of cell death induced at a determined radiation dose (β = ΔRIA/Δln(Gy)). Higher β values (increased rate of RIA at given radiation doses) were observed in patients with low sexual toxicity (Exp(B) = 0.83, C.I. 95% (0.73-0.95), p = 0.007; Exp(B) = 0.88, C.I. 95% (0.82-0.94), p = 0.001; Exp(B) = 0.93, C.I. 95% (0.88-0.99), p = 0.026 for 24, 48 and 72 hours respectively). This relation was also found with rectal (Exp(B) = 0.89, C.I. 95% (0.81-0.98), p = 0.026; Exp(B) = 0.95, C.I. 95% (0.91-0.98), p = 0.013 for 48 and 72 hours respectively) and urinary (Exp(B) = 0.83, C.I. 95% (0.71-0.97), p = 0.021 for 24 hours) toxicity.

**Conclusion:**

Radiation induced apoptosis at different time points and radiation doses fitted to a semi logarithmic model defined by a mathematical equation that gives an individual value of radiosensitivity and could predict late toxicity due to radiotherapy. Other prospective studies with higher number of patients are needed to validate these results.

## Background

Interpatient heterogeneity in normal tissue reactions varies considerably, yet the genetic determinants and the molecular mechanisms of therapeutic radiation sensitivity remain poorly understood [1]. Patients treated with radiotherapy (RT) will develop clinical toxicity and this may limit the efficacy of the treatment [2]. Even with rigid dose tolerance limits, patients respond with different levels of toxicity to a given RT schedule [3]. The treatment of cervical carcinoma includes surgery and/or irradiation. The prediction of the toxicity induced by RT could help to select the most appropriate treatment for each patient. Many predictive factors of tumour radiosensitivity have been described, most of them related to gene expression patterns [4,5]. Intrinsic radiosensitivity is correlated to the ability of the cell to detect and repair DNA damages [6]. Flow cytometry evaluation of lymphocyte apoptosis has been established as a reliable method to measure radiation-induced damage [7]. Quantification of radiation-induced apoptosis (RIA) in peripheral blood lymphocytes (PBLs) has been proposed as a possible screening test for cancer-prone individuals and also for the prediction of normal tissue responses after RT [8]. Previous reports have suggested that PBLs apoptosis could be used to identify radiosensitive patients based on the apoptotic response of T lymphocytes to large in vitro doses [9]. In this way, radiation-induced T-lymphocyte apoptosis can significantly predict differences in late toxicity between individuals [10]. A correlation existed between low levels of RIA in lymphocytes and increased late toxicity after radiation therapy. The radiation-induced apoptotic responses of the CD4 and the CD8 T-lymphocytes from both groups of hypersensitive patients are significantly lower than the responses of the CD4 and the CD8 T-lymphocytes from normal individuals [7]. Moreover, apoptosis in subpopulations of T lymphocytes (CD4+ and CD8+) could be used to identify radiation-sensitive patients before therapy [10]. Development of predictive assays for clinical prediction requires that the diagnostic test employed display both high reproducibility and low variation [11]. The ideal pre-RT predictive assay must be cheap, fast, with low false positives or negatives results and accessible for clinical implementation. Intrinsic radiosensitivity is genetically determined and varies in dependence of the patient and the tumour type. For this, the aim of the present study was to define a radiosensitivity value for each individual patient and to test if such marker of apoptosis will predict the risk of late toxicity in cervical cancer patients treated with radiotherapy in a prospective assay.

## Methods

### Patients

Ninety four consecutive patients with histological confirmed localized carcinoma of the uterine cervix, diagnosed and treated in our institution between February 1998 and October 2003, and given inform consent, were recruited prospectively for the study. Apoptosis was determined between April 2003 and March 2004. The study was approved by the Research and Ethics Committee of our institution. Mean age of patients was 51.12 ± 13.18 years (range 26-89). Evaluation of clinical toxicity was made, and the mean follow-up was 26.14 ± 17.61 months (range 3-73). The majority of patients had squamous cervical carcinoma (76 cases; 76.8%) in early stage of the disease (I (52.68%)-II (40.20%)). Characteristics of patients are detailed in Table 1. A total dose of 45-50 Gy was administered in a 1.8-2 Gy/day schedule followed by brachytherapy treatment at a dose of 20 Gy to a total dose of 69.39 ± 15.28 Gy. Sexual, bowel, rectal and urinary toxicity were evaluated according to the LENT SOMA system for recording late effects [12], stratifying toxicity reaction from 0 (no toxicity) to 4 (serious toxicity). Clinical toxicity of patients is detailed in Table 2. Four healthy donors were included as controls. All samples were processed anonymously.

**Table 1:** Characteristics of the patients in study (n = 94)

	Cases	Percentages
**Stage**		
I	51	52.6
II	40	41.2
III	5	15.2
IVA	1	1.0
**Histology**		
Squamous	76	76.8
Adenocarcinoma	23	23.2
**Grade**		
I	11	13.1
II	41	48.8
III	32	38.1
**Treatment**		
Surgery + RT (posterior)	22	22.2
Surgery + RT (posterior) + Chemotherapy	7	7.1
Radical RT	19	19.2
Radical RT + Chemotherapy	51	51.5

**Table 2:** Toxicity of 94 cervical cancer patients

Toxicity	Grade 0	Grade 1	Grade 2	Grade 3	Grade 4
Sexual	15 (16.0%)	31(33.0%)	32 (34.0%)	12 (14.9%)	2 (2.1%)
Bowel	67 (71.3%)	21 (22.3%)	3 (3.2%)	2 (2.1%)	1 (1.1%)
Rectal	70 (74.5%)	11 (11.7%)	9 (9.6%)	3 (3.2%)	1 (1.1%)
Urinary	67 (71.3%)	16 (17.0%)	8 (8.5%)	2 (2.1%)	1 (1.1%)

### Sample collection

A total of 10 ml of blood was collected into lithium heparin venous blood collection tubes (Vacutainer, BD Biosciences, San Jose, CA). PBLs were isolated by density gradient centrifugation on Ficoll-Hypaque (Lymphoprep, Gybco, Life Technologies, Grand Island, NY, USA). Cells were suspended in RPMI 1640 medium (0.05% L-glutamine, 20 nM HEPES, 50 IU/ml penicillin, 50 ug/ml streptomycin and 10% heat inactivated fetal calf serum (FCS)). The final concentration of cells was adjusted to 2 × 10^5 ^cells/ml in complete RPMI, and they were separated into four 25-cm^2 ^flasks (three flasks for irradiation and one control flask) with 5 ml of medium.

### Sample irradiation and preparation

Cells were irradiated at room temperature with 1, 2 and 8 Gy, 6 mV X rays (Mevatron, Siemens, Germany) at a dose rate of 50 cGy/min. After irradiation, the preparations were incubated at 37°C in 5% CO_2 _during 24, 48 or 72 hours. Post incubation, four samples of 1.5 × 10^5 ^cells from each flask (one negative control and three samples for triplicate study) were placed into 5-ml centrifuge tubes and washed once with 3 ml of PBS free of Ca^2+ ^and Mg^2+^. The tubes were centrifuged at 500 g for 10 min and the supernatant was removed. Cells were incubated with 5 μl of monoclonal antibody CD45 APC-conjugated monoclonal antibody, permitting the exclusion of erythrocytes, debris, and leukocytes (clone HI30, Pharmingen, Becton Dickinson, San José, CA, USA).

### Apoptosis assay

The apoptosis analysis was determined by Annexin V kit (Pharmingen, Becton Dickinson, San José, CA, USA) according to manufactor instructions. The supernatant was decanted and the pellet was resuspended in 100 μl of 1× annexin V binding buffer. Cells were incubated with 4 μl of annexin V-FITC and 10 μl of propidium iodide (PI) for 15 minutes at room temperature in the dark. Finally, 400 μl of 1× annexin V Binding Buffer was added.

### Flow cytometry

Flow cytometric analyses were performed on a FACScalibur flow cytometer (BD, San Jose, CA) equipped with an argon-ion laser. Each sample was analyzed using 5000 events/sample acquired in list mode by a Macintosh Quadra 650 minicomputer (Apple computer Inc., Cupertino, C). Data analysis was performed via three-step procedure using the Cellquest software (BD, San Jose, CA). Apoptosis levels were measured at four radiation doses (0, 2, 4, and 8 Gy) in triplicate. Detection of phosphatidyl serine exposure on the external leaflet of the plasma membrane employing annexin V was assayed. This phenomenon is one of the early events in the cascade of events characterizing apoptosis.

### Statistical analyses

Statistical analyses were performed using the SPSS Statistical Package (version 15.0 for Windows). χ^2 ^test was used to compare discrete variables. ANOVA and Kruskall-Wallis test were used to compare continuous variables. Cox regression was used for multivariate analysis. Kolmogorov-Smirnoff analysis was made to determine the distibution of data. All tests were two sided and values of p < 0.05 were considered to be statistically significant.

## Results

### A mathematical model predicts toxicity due to radiotherapy

**Figure 1:**
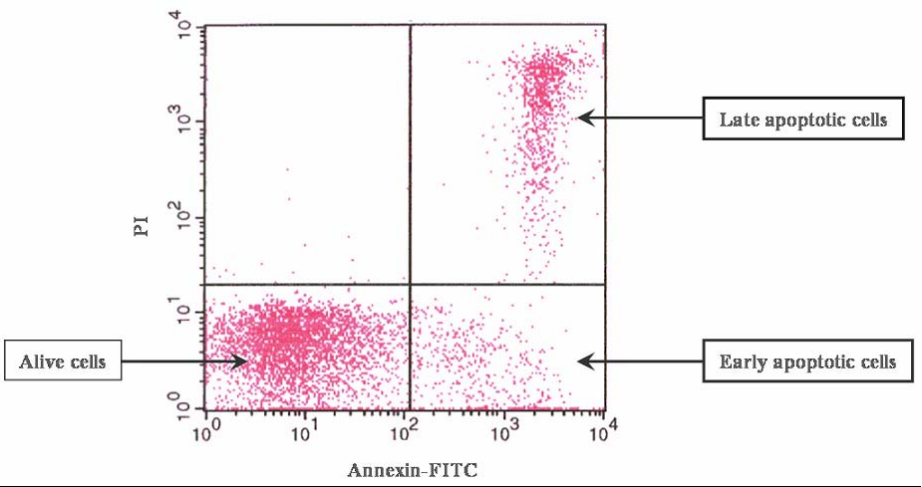
**Determination of apoptosis**. Three different cell populations were detected after the Annexin V/PI staining of isolated PBLs. Alive cells grouped in the lower left part of the panel, early apoptotic cells grouped in the lower right part of the panel and late apoptotic cells grouped in the higher right part of the panel.

**Figure 2:**
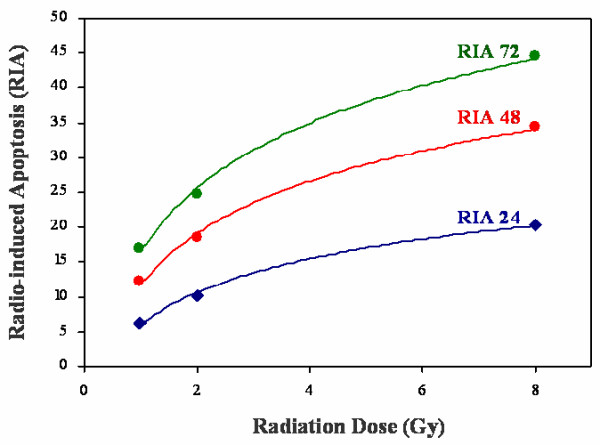
**Radio-induced apoptosis (RIA) of lymphocytes after 24, 48 and 72 hours**. RIA values at 1, 2 and 8 Gy were adjusted perfectly to a semi logarithmic model where two constants were defined: α is the origin of the curve in the Y axis and determines the percentage of spontaneous cell death and β is the slope of the curve and determines the percentage of cell death induced at a determined radiation dose (β = ΔRIA/Δln(Gy)).

After irradiation of lymphocytes, three different populations of PBLs were observed: i) early apoptotic cells identified with annexin V, ii) late apoptotic cells stained with propidium iodide (PI) and iii) non-apoptotic cells (Figure 1). Radio-induced apoptosis (RIA) could be defined as the percentage of total PBLs death induced by the radiation dose minus the spontaneous cell death (control, 0 Gy). RIA data followed a normal distribution (Table 3). RIA values increased with radiation dose (0, 1, 2 and 8 Gy), and fitted to a semi logarithmic equation as follow: RIA = βln(Gy) + α (Figure 2). Alpha (α) is defined as the origin of the curve in the Y axis and determines the percentage of cell death at no radiation dose (spontaneous apoptosis). Beta (β) is defined as the slope of the curve and determines the percentage of cell death induced at a determined radiation dose (β = ΔRIA/Δln(Gy)). β seems to represent an individual marker of radiosensitivity. RIA increased significantly with radiation dose and incubation time. The α value raised with the incubation time (p < 0.001) while the β value did not (Table 4). The adjustment coefficients (R) were determined and data strongly fitted to a semi logarithmic mathematical model, with correlation values of 0.99 for 24 and 48 hours and 0.98 for 72 hours (Table 4). β values of 24 h (11.19 ± 4.18), 48 h (9.48 ± 4.40) and 72 h (10.22 ± 5.39) were closely statistically correlated using the Pearson test (24 vs. 48 h, p < 0.001; 24 vs. 72 h, p = 0.014; 48 vs. 72 h, p < 0.001). Intraindividual variation for β value for healthy donors was lower than interindividual variation for patients (14.30% vs. 45.49%).

**Table 3:** Data of apoptosis and radio-induced apoptosis (RIA) of PBLs treated with 0, 1, 2 and 8 Gy of radiation at 24, 48 and 72 hours. Cells isolated from 94 cervical cancer patients. Mean ± SD was included. RIA data followed a normal distribution (Kolmogorov-Smirnoff test, p = NS) and strongly fitted to a semi logarithmic model

	Apoptosis			Radio-induced apoptosis (RIA)		
		
Dose (Gy)	24 h	48 h	72 h	24 h	48 h	72 h
0	25.32 ± 21.31	28.33 ± 21.30	24.03 ± 19.13			
1	31.88 ± 20.24	40.29 ± 20.34	41.58 ± 21.20	6.29 ± 5.39	12.23 ± 7.67	17.02 ± 9.79
2	35.52 ± 20.78	46.68 ± 20.74	49.18 ± 21.78	10.13 ± 6.72	18.36 ± 9.78	24.68 ± 11.87
8	46.02 ± 21.11	62.63 ± 20.44	69.09 ± 19.06	20.44 ± 11.47	34.30 ± 15.06	44.48 ± 17.59

**Table 4:** α and β constants and adjustment coefficients (R) at 24, 48 and 72 hours

Time (hours)	α (mean ± SD, median, range)	β (mean ± SD, median, range)	R (mean ± SD, median, range)
24	14.50 ± 9.69, 15.42 (-29-46.7)	11.19 ± 4.18, 11.19 (-5.42-23.70)	0.94 ± 0.11; 0.99 (0.29-1)
48	25.84 ± 9.99, 26.22 (-5.07-44.44)	9.48 ± 4.40, 9.34 (-8.00-19.83)	0.96 ± 0.068, 0.99 (0.64-1)
72	28.46 ± 10.76, 30.31 (-0.46-51.32)	10.22 ± 5.39, 10.75 (0.24-32.83)	0.93 ± 0.15, 0.98 (0.11-1)

### RIA predicts radio-induced toxicity. Role of α and β constants

Sexual, bowel, rectal and urinary toxicity were evaluated according to the Lent-Soma system for recording late effects (Table 2). The majority of patients did not suffer toxicity or suffered low grade of toxicity, especially for gastrointestinal (71.3%), rectal (74.5%) and urinary damage (71.3%). Half of the population in study (48 cases; 51.1%) maintained sexual relations when the analysis were performed. A simple Cox regression analysis or proportional hazards model was performed to evaluate the relationship between β and the different normal tissue toxicity reactions observed. The hazard ratio was also estimated (Table 5). Rectal, urinary and sexual toxicities were significantly predicted by the β constant at different time points. PBLs apoptosis, measured as an integrated value of radiosensitivity (from 1 to 8 Gy), seems to have the potential to predict which patients will be spared late toxicity after radiation therapy. Patients with late toxicity after radiotherapy showed lower lymphocyte apoptotic responses than patients who had not developed late toxicity.

**Table 5:** Correlation between β constant at 24, 48 and 72 hours with late toxicity. Simple Cox regression analysis. p value, Exp(B) and C.I. 95% were included. *: grades 0 vs. 1-4, #: grades 0-2 vs. 3-4

Constant	Bowel Toxicity *	Rectal Toxicity *	Urinary Toxicity *	Sexual Toxicity # /**Sexual relations**
β24	p = 0.073 0.916 (0.832-1.008)	p = 0.063 0.908 (0.821-1.005)	p = 0.021 0.835 (0.717-0.973)	p = 0.007 0.834 (0.730-0.951)
β48	p = 0.071 0.914 (0.830-1.008)	p = 0.026 0.897 (0.816-0.987)	p = 0.053 0.855 (0.729-1.002)	p = 0.001 0.884 (0.828-0.944)
β72	p = 0.834 0.984 (0.843-1.147)	p = 0.013 0.951 (0.913-0.989)	p = 0.376 0.946 (0.837-1.069)	p = 0.026 0.935 (0.882-0.992)

## Discussion

Normal tissue toxicity due to radiotherapy (RT) limits the efficacy of the treatment. The development of predictive toxicity assays would improve RT results. Analysis of radiation induced apoptosis (RIA) in peripheral blood lymphocytes (PBLs) by flow cytometry seems to be a useful approach to determine individual variability to RT. The present study suggests a new approach to the evaluation of individual radiosensitivity scored by flow cytometry. A mathematical model where RIA and late toxicity were related at different radiation doses and time points was developed. We observed that radiation-induced apoptosis of lymphocytes (constant β) varied between patients, representing an intrinsic condition of each individual. β value predicted all radiation toxicities evaluated but bowel morbidity. This fact could be explained by the lower dose of radiation given in the bowel compared with rectum and bladder [13]. This higher dose received in these organs caused higher number of patients with toxicity that can be evaluated (Table 2). Bowel toxicity is infrequent and mild compared with other toxicities [14]. Sexual difficulties after treatment for gynaecological malignancy have received increased attention in recent years. Women receiving primary or adjuvant pelvic radiotherapy experienced greater and more prolonged disruption to their sexual well-being [15]. In fact, sexual toxicity was predicted by β values at 24, 48 and 72 hours. Higher levels of β values were significantly associated to lower levels of late toxicity. This finding agree with previous studies [7,16] where RIA presented higher levels in healthy patients compared with radiosensitive patients and patients who suffered ataxia-telangiectasia (AT) [9]. This profile was observed in different lymphocyte subpopulations [10,17]. Different molecular and genetic alterations could help to explain those findings; however the mechanism behind the relationship between increased radiation toxicity and reduced apoptotic response in PBLs is still unclear. Lymphocytes from patients who suffered AT, Bloom syndrome, Fanconi anaemia and other syndromes related with radiosensitivity, showed abscense of induction of p53 [18,19] and lower levels of Bax [20]. This failure in the induction of the apoptosis response in lymphocytes seems to be based on molecular alterations and has been related with late toxicity [10]. Only two studies did not find a relation between radiation-induced apoptosis in PBL and toxicity [8,21]. Differences related to the experimental model could explain these findings. DNA radio-induced damage seems to be independent of the final effect in the cell. A great variability in the signalling and repair mechanisms due to radio-induced cell damage must be presented. Differences in gene expression profiles seem to have a relevant role in this variability [4,5]. Moreover, certain single nucleotide polymorphisms located in candidate genes associated with the response of cells to radiation (i.e. ATM, SOD2, XRCC1, XRCC3, TGFB1 or RAD21) have been suggested as important factors for the development of late radiation toxicity [22]. In summary, the present study is characterized by the development of a mathematical model that defines an integrated value of the intrinsic radiosensitivity observed at different radiation doses in each patient. The RIA values through different experiments strongly fitted to a semi logarithmic model. The β constant, that defines the individual radiosensitivity and constitutes the predictive value, need extensive and more prospective studies to be validated.

## Conclusion

In our opinion, it is possible to estimate the cellular radiosensitivity of PBLs of patients analyzing the RIA rate by annexin V/PI staining flow cytometric analysis. We were able to define an intrinsic individual value of radiosensitivity (β constant) integrating the apoptotic response at increasing radiation dose. This β constant does not change with different incubation times and it could represent a novel predictive parameter for clinically induced radiation toxicity. Feasibility and cost effectiveness of this assay would favour larger studies to analyze the predictive role of this model, especially in different lymphocyte subpopulations.

## List of abbreviations

AT: Ataxia-Telangiectasia; PBLs: Peripheral Blood Lymphocytes; PI: Propidium Iodide; RIA: Radio-induced Apoptosis; RT: Radiotherapy.

## Competing interests

The authors declare that they have no competing interests.

## Authors' contributions

EB has written the first draft of the manuscript, has been involved in conception and design of the project and has made all the cell experiments with lymphocytes, irradiation of cells, flow cytometry experiments, data acquisition and statistical analysis. LAHH has written the manuscript, has made tables and figures and has been involved in type of packaging likewise in the submission process. PCL has been involved in conception and design of the study and in drafting the manuscript and has given final approval of the version to be published. BP and MLl have made the selection of patients, the evaluation of clinical variables and grade of toxicity as well as all the aspects related with the patients selected, including the treatment. FF has participated in cell experiments, irradiation of cells and flow cytometry experiments. CRG has been involved in flow cytometry experiments as well as in RIA measurements. All authors read and approved the final manuscript.
